# Assessment of radiological hazards arising from natural radioactivity in Bayburt stones used in buildings and possible effects on public health

**DOI:** 10.3389/fpubh.2026.1821213

**Published:** 2026-04-23

**Authors:** Serdar Dizman, Muttaka Umar, Yavuz Selim Hatipoglu

**Affiliations:** 1 Department of Physics, Faculty of Arts and Sciences, Recep Tayyip Erdogan University, Rize, Türkiye; 2 Department of Physics, Institute of Graduate Studies, Recep Tayyip Erdogan University, Rize, Türkiye; 3 Department of Civil Engineering, Faculty of Engineering, Bayburt University, Bayburt, Türkiye; 4 Department of Occupational Health and Safety, Graduate School, Avrasya University, Trabzon, Türkiye

**Keywords:** Bayburt, mining site, natural stone, radioactivity, risk assessment

## Abstract

**Background:**

Since ancient times, natural stones have served as essential materials in construction, landscaping, and artistic applications. Bayburt stone is one of the most important stones used in and around Bayburt, in the Eastern Black Sea region of Türkiye. It has been frequently used in buildings (castle, house, etc.), street flooring, fountains, and places of worship from ancient times to the present day. Due to their underground origin, natural stones contain natural radioisotopes in various concentrations, making the assessment of their radioactivity levels important for public health.

**Objective:**

This study aims to determine the natural radioactivity levels of Bayburt stones used in buildings and to assess the potential radiological hazards and possible effects on public health.

**Methods:**

Natural radioactivity levels (^226^Ra, ^232^Th and ^40^K) were determined in a total of 36 Bayburt stone samples, 30 from five different quarries and 6 from a natural stone factory in Bayburt province. High-purity germanium (HPGe) spectrometry was employed to measure radioactivity concentrations. Additionally, several radiological hazard indices (Ra_eq_, H_in_, I_γ_, D, AEDE, AGDE, and ELCR) were calculated and compared with reference limits proposed by international organizations.

**Results:**

The radioactivity concentrations in the Bayburt stone samples ranged from 1.56 to 23.54 Bq/kg for ^226^Ra, from 0.53 to 23.69 Bq/kg for ^232^Th, and from 10.31 to 505.35 Bq/kg for ^40^K. The average activity concentrations were found to be 5.56 ± 0.90 Bq/kg for ^226^Ra, 4.62 ± 0.65 Bq/kg for ^232^Th, and 179.12 ± 9.88 Bq/kg for ^40^K. The average values of Ra_eq_, H_in_, I_γ_, D, AEDE, AGDE, and ELCR were found to be 18.68 Bq/kg, 0.07, 0.14, 8.91 nGy/h, 43,72 μSv/y, 63.06 μSv/y, and 17.07 × 10^−5^, respectively. All calculated radiological hazard indices were below the recommended safety limits.

**Conclusion:**

The findings indicate that the Bayburt stone samples investigated are suitable for construction purposes and do not pose significant radiological hazards to human health when used in building environments.

## 1 Introduction

Bayburt stone is a widely used natural building material in northeastern Türkiye due to its favorable mechanical properties, aesthetic appearance, and local availability (1). The stone is extensively employed in residential and commercial construction, including wall cladding, flooring, decorative facades, and structural components, and its durability, workability, and resistance to weathering make it economically advantageous compared to imported materials (2). The abundant quarry reserves in the Bayburt region further enhance its accessibility and cost-effectiveness for the local construction industry (3). These characteristics have contributed to its continued demand in both urban and rural construction projects.

Geologically, Bayburt stone occurs as tuffite layers composed of Eocene volcano-sedimentary rocks that unconformably overlie Jurassic metamorphic and carbonate units. Its low hardness, fine-grained texture, and ease of processing increase its practical appeal, and studies conducted by the Mineral Research and Exploration (MTA) have estimated its block yield at approximately 2.5 million tons, with probable and possible reserves of 650,000 and 600,000 tons, respectively (2). The long-standing use of this stone in regional architectural structures reflects its availability and workability within the area (4, 5). With the establishment of a modern stone processing facility in Bayburt, the material is now marketed both domestically and internationally, contributing to regional economic activity (5).

The use of materials containing naturally occurring radioactive materials (NORMs) in construction may pose health risks due to prolonged exposure to gamma radiation and radon gas (6). The greatest contribution to the effective dose received through inhalation comes from Rn-222, a decay product of radium in the uranium series. Rn-222 is a colorless and odorless gas that can accumulate in enclosed spaces, posing potential for serious health risks. Therefore, monitoring radon levels and prioritizing ventilation systems are recommended for buildings constructed with naturally derived building materials such as Bayburt stone. The concentration of NORMs in building stones is largely governed by their geological origin and mineralogical composition (7). Radionuclides such as ^226^Ra, ^232^Th, and ^40^K are typically associated with specific mineral phases, including feldspar and heavy mineral fractions (8). Variations in rock type, density, mineral content, and geochemical evolution may therefore influence the natural radioactivity levels observed in construction materials (9, 10).

Prolonged exposure to these radionuclides through indoor gamma radiation and radon exhalation may increase radiological health risks (11). To regulate public exposure, international bodies such as the United Nations Scientific Committee on the Effects of Atomic Radiation (UNSCEAR) and the European Commission (EC) have established recommended safety limits for radiation exposure. The recommended annual effective dose limit for members of the public is 1 mSv/y (12), and reference values such as radium equivalent activity (370 Bq/kg) and activity concentration index thresholds are commonly used in evaluating building materials (13).

Despite the widespread use of Bayburt stone in construction, no published studies have reported baseline data on the activity concentrations of key radionuclides (^226^Ra, ^232^Th, and ^40^K) in this material. The absence of such data represents a gap in the radiological characterization of locally sourced building stones in northeastern Türkiye. Establishing these baseline concentrations is essential for assessing potential radiological hazards associated with their application in residential and commercial structures.

Therefore, this study aims to quantify the concentrations of natural radionuclides in Bayburt stone and to evaluate the associated radiological hazard indices commonly used in building material assessments. Previous investigations conducted in other regions have applied gamma spectrometry techniques to determine radionuclide concentrations and assess hazard parameters such as absorbed dose rate, activity concentration index, alpha index, and annual effective dose (14–16).

## 2 Methods

### 2.1 Samples collection and preparation

**Figure 1:**
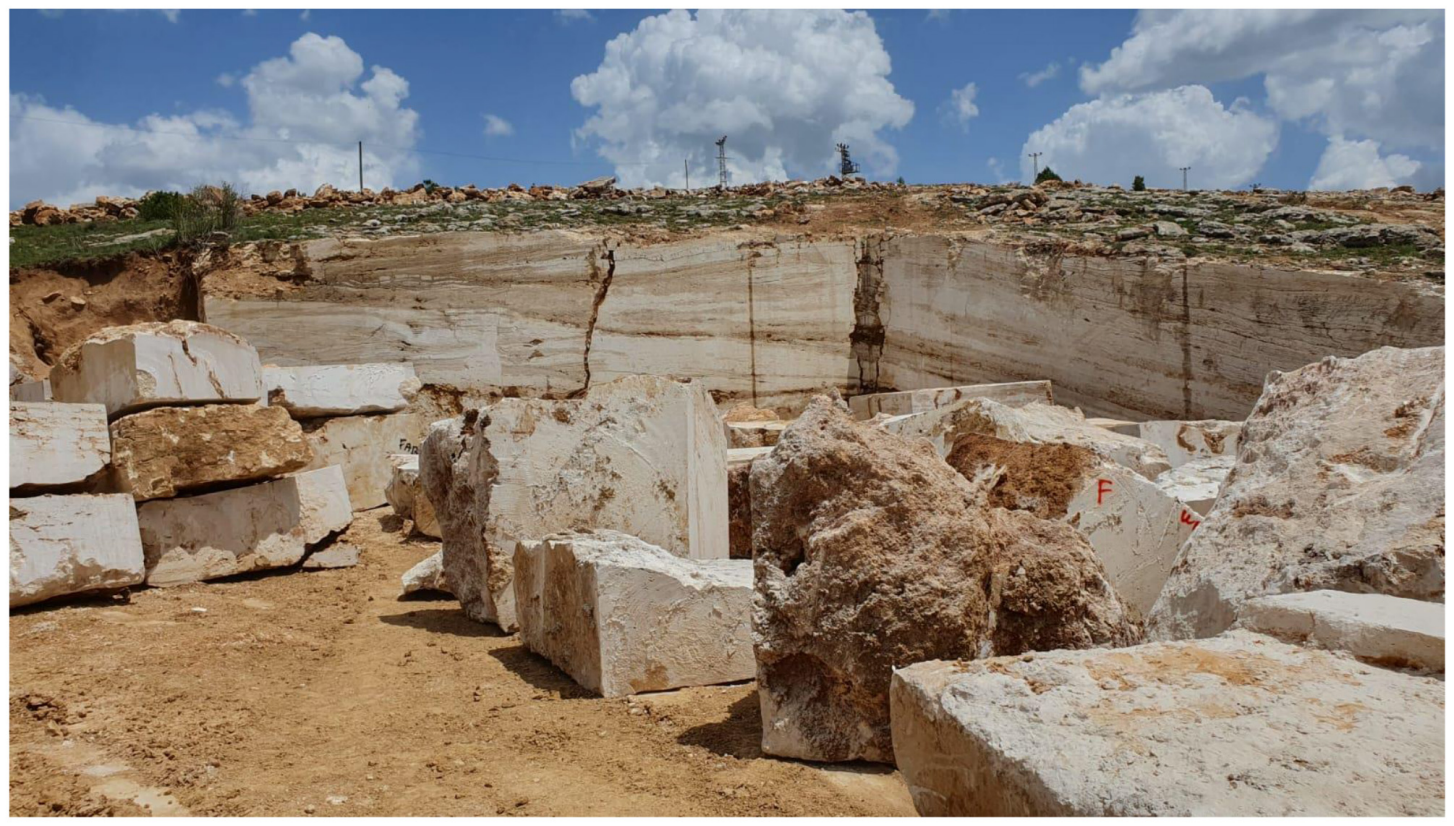
An image from one of the Bayburt mining sites where the samples were taken.

**Figure 2:**
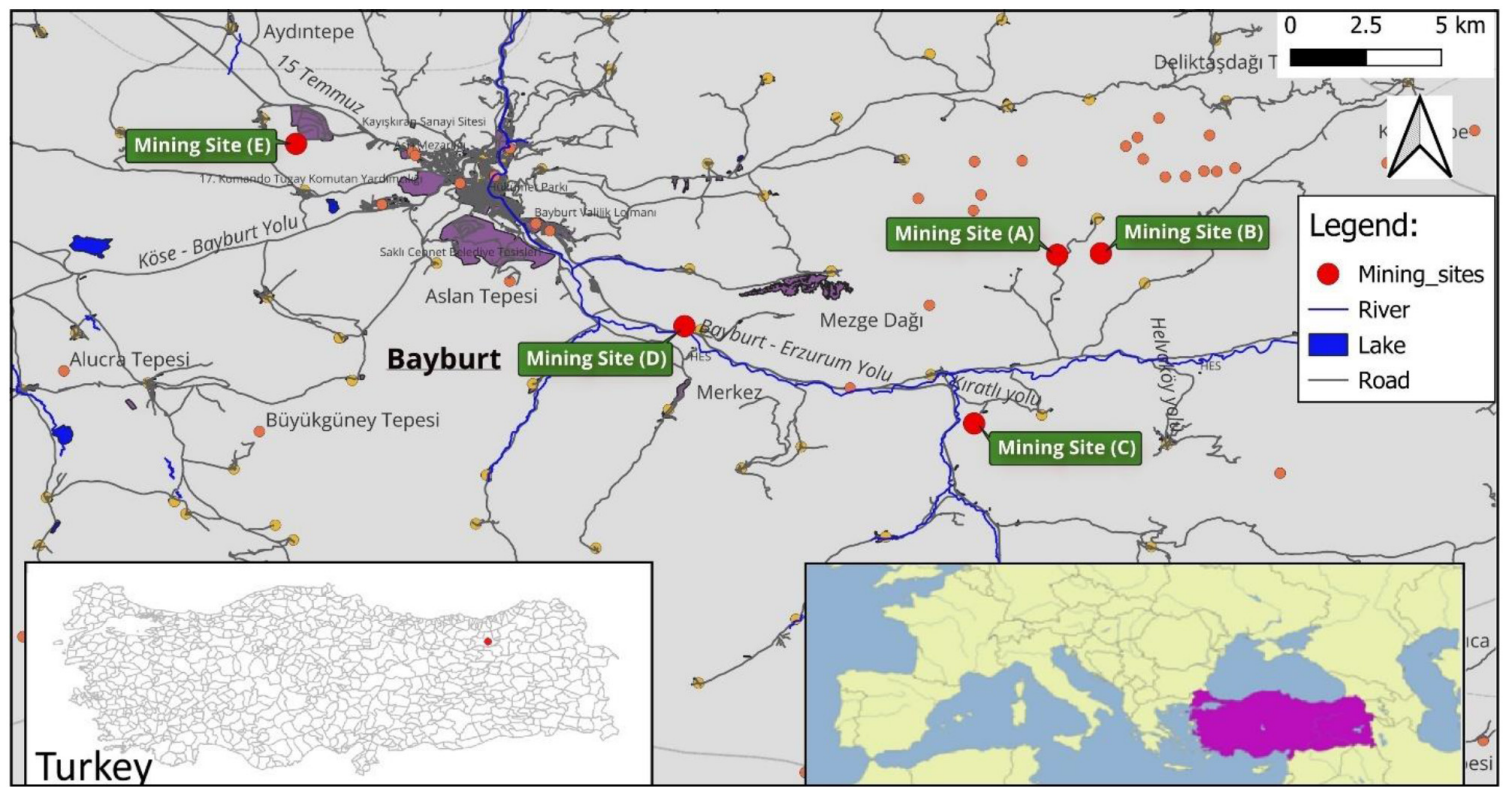
General map showing the location of the mining sites in Bayburt province.

**Figure 3:**
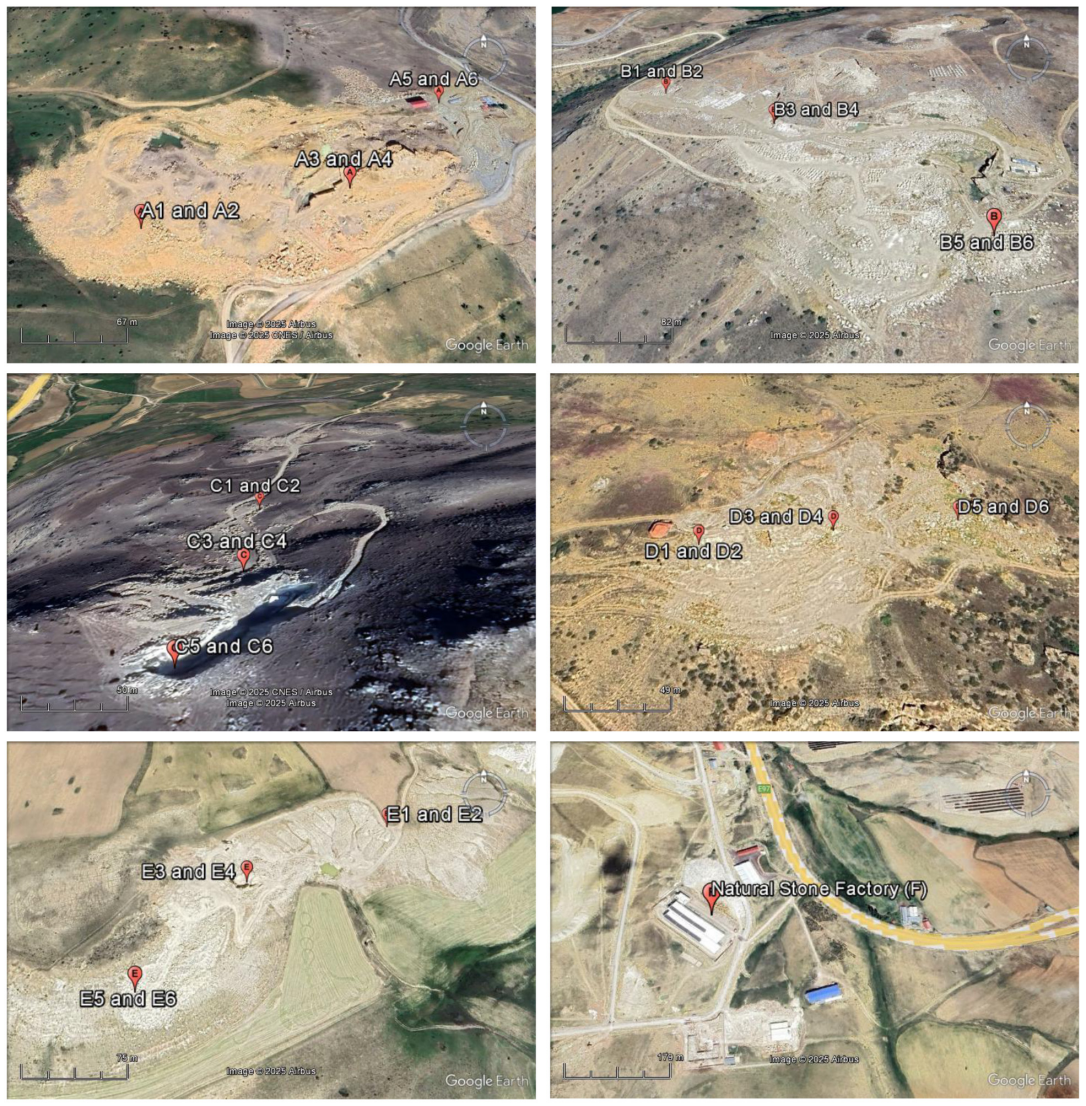
Map showing the coordinates where stone samples were collected based on mining areas and the natural stone factory where end-user samples were taken.

**Figure 4:**
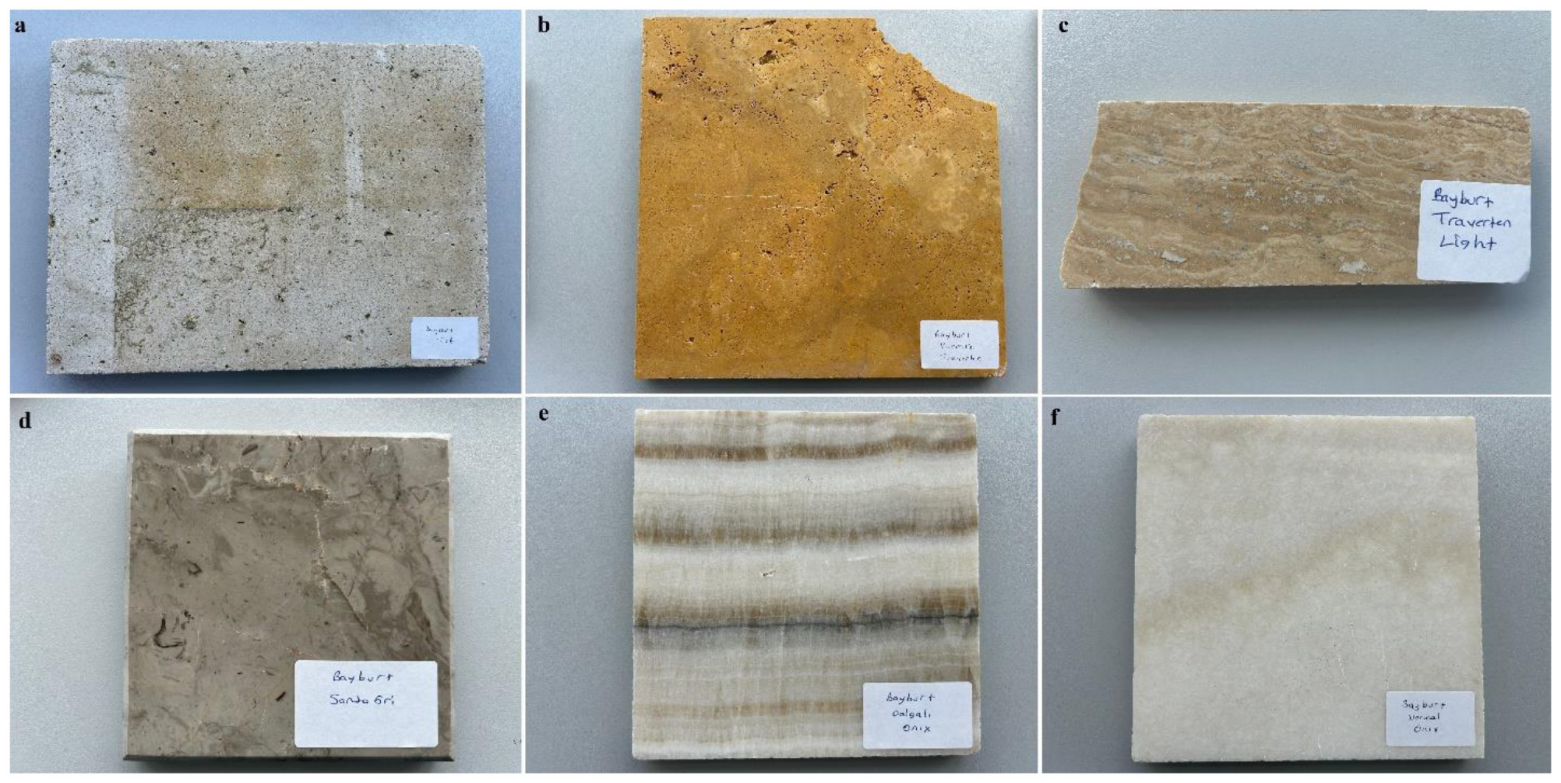
Ready-made Bayburt stone samples taken from Natural Stone Factory [**(a)** Bayburt Tüf; **(b)** Bayburt Kirmizi; **(c)** Bayburt Traverten Light; **(d)** Bayburt Santa Gri; **(e)** Bayburt Dalgali Onix; **(f)** Bayburt Normal Onix].

A total of 36 samples and their derivatives were collected using a systematic approach to ensure a representative dataset for the Bayburt region. Thirty of the samples were collected from 5 Bayburt stone mines in the Bayburt province and 6 processed and ready-for-sale samples were taken from the Bayburt Natural Stone Factory. The samples (F1–F6) examined as derivatives of Bayburt stone were Bayburt Tüf, Bayburt Kirmizi, Bayburt Traverten Light, Bayburt Santa Gri, Bayburt Dalgali Onix, and Bayburt Normal Onix. Figure 1 shows an image of one of the mining sites from which Bayburt stone samples were collected. The mining sites (A-E) were chosen to capture the geological diversity of the Eocene-aged basins. At each site, both surface and subsurface samples were collected to identify any variations in radionuclide concentration through the vertical profile of the quarry. Surface samples were collected at three points: the entrance, middle, and exit of each mine area, to represent the geological diversity of each mining site. Subsurface samples were collected at a depth of approximately 1 meter, directly below the surface sampling points. Figures 2, 3 show maps with the coordinates and locations where the samples were collected based on mining areas and where end-user samples were taken from the stone factory. The images of the six Bayburt Natural Stone Factory end-user product samples taken are shown in Figure 4. The Bayburt stone samples were taken to the Nuclear Physics Research Laboratory at Recep Tayyip Erdogan University for analysis. The stone samples were initially crushed into smaller fragments with a sledgehammer in the laboratory. These fragments were then pulverized into fine powder with a grinding mill (Zhonghe, Model ZHM-1T). The resulting powder was transferred into clean cylindrical plastic containers of uniform 100 mL size. Each container was tightly sealed with parafilm to ensure it remained airtight. Prior to measurement, the samples were kept and stored for 4 weeks to ensure that radium and its short-lived decay products attained secular equilibrium (17).

### 2.2 Radioactivity measurements

**Figure 5:**
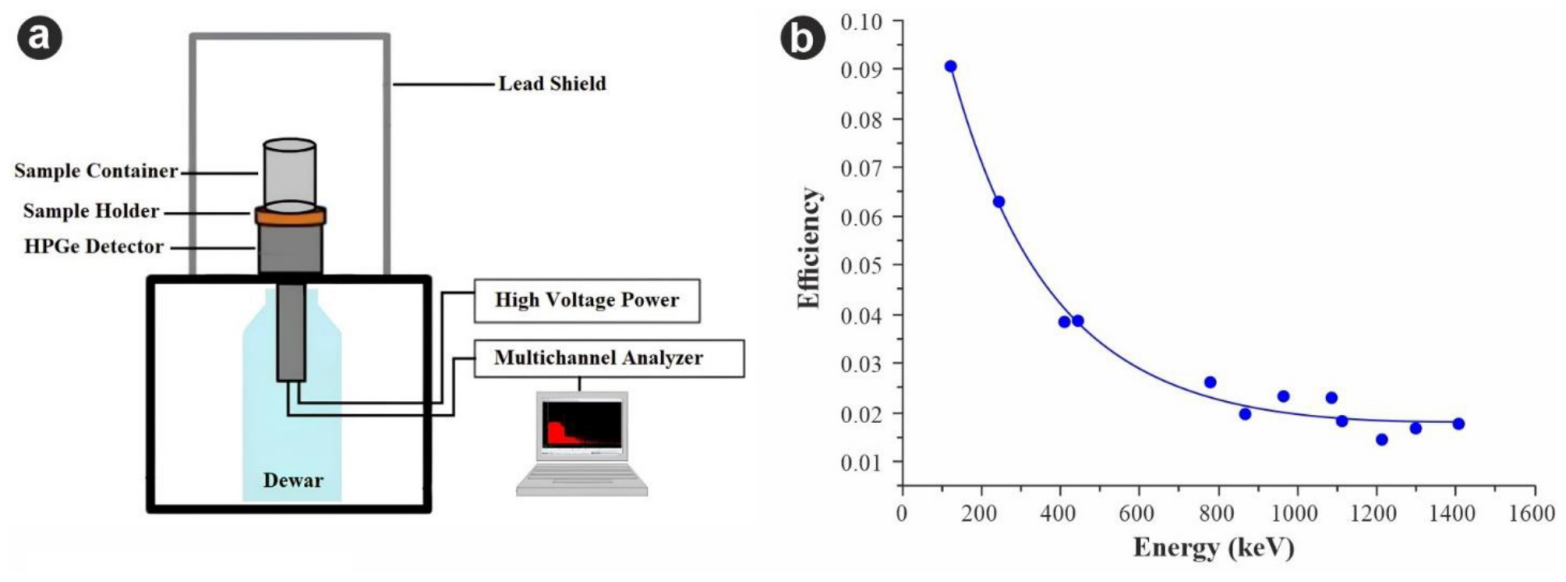
**(a)** The experimental scheme of the gamma spectrometer system. **(b)** The efficiency curve obtained from Eu-152 standard source.

Measurements of Radium-226, Thorium-232, and Pottasium-40 activity concentrations were performed with a high-purity germanium (HPGe) detector (ORTEC HPGe, Model No; GEM55P4-95, 55% relative efficiency) with a resolution (FWHM) of 1.9 keV at 1.332 MeV gamma ray of ^60^Co. The detector was enclosed within a 10 cm-thick lead shield to minimize background radiation levels. With efficiency being one of the most fundamental features of detectors, ^152^Eu with known activity was used for the detector efficiency determination because of their bigger energy range (122, 244, 344, 411, 443, 779, 964, 1,112, and 1,408 keV) with emission probabilities ranging from 3 to 29 % (18). High-Purity Germanium (HPGe) spectrometry was employed for its superior energy resolution, which is critical for identifying low-level radionuclides in natural materials (19). This technique allows for the precise separation of gamma-ray peaks, ensuring a more accurate quantification than broader-spectrum methods like NaI(Tl) detectors (20). An ideal measurement setup was established using cylindrical source geometry characterized by uniform activity distribution, constant volume, and a fixed detector-to-source distance. The source was positioned directly in line with the detector to determine efficiency. Figure 5a illustrates the experimental setup of the gamma spectrometer used in this study (21). The efficiency curve obtained for the detector is shown in Figure 5b. Comprehensive details regarding the efficiency calibration of the detector employed in this study are provided in a previously published work (22).

**Figure 6:**
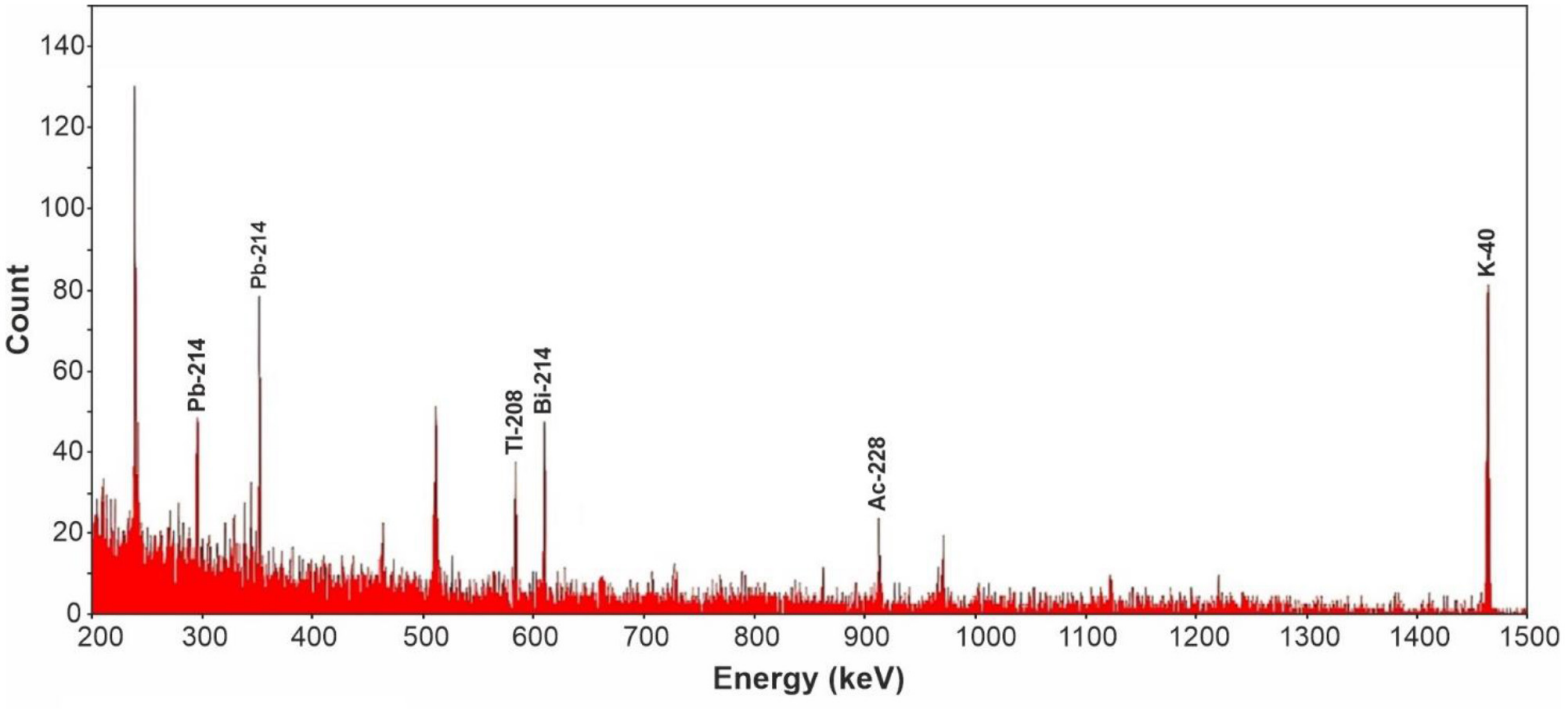
A typical spectrum image obtained from measuring samples at the detector.

The ^226^Ra activity concentration was calculated from the gamma emissions of its decay daughters, ^214^Pb (295 and 351 keV) and ^214^Bi (609 and 1,120 keV). The activity concentration of ^232^Th was estimated through the correlation with the measured activities of its decay daughters, namely ^214^Pb (238 keV), ^228^Ac (338 keV and 911 keV), and ^208^Tl (583 keV) gamma-ray energies. Determination of ^40^K activity concentrations was based on its distinct gamma emission at 1,460 keV (23). The samples were individually placed above the detector and counted for 50,000 seconds, with a sample spectrum illustrated in Figure 6. Spectra were analyzed using Gamma Vision (Version 8.0, AMETEK Inc., Tennessee, USA) software, and activity concentrations were computed after accounting for background radiation and detector efficiency.

In order to remove background contributions from measurements, empty containers were analyzed using identical measurement conditions as those applied to the samples. After the background measurement and extraction, radionuclide activity concentrations were determined according to Equation 1.


$$\begin{array}{l} A\left(Bq/kg\right)=\frac{C}{\epsilon P_{\gamma }mt} \end{array}$$ (1)

where C is the net peak area, ϵ is the detector efficiency, *P*_γ_ is the gamma emission probability, *m* is the mass of the sample (kg) and *t* is the counting time (s). The uncertainty (ΔA) of the radioactivity concentrations was calculated using Equation 2 (17).


$$\begin{array}{l} \Delta A=A\sqrt{{\left(\frac{\Delta C}{C}\right)}^{2}+{\left(\frac{\Delta \varepsilon }{\varepsilon }\right)}^{2}+{\left(\frac{\Delta P}{P}\right)}^{2}+{\left(\frac{\Delta m}{m}\right)}^{2}} \end{array}$$ (2)

where ΔC indicates the uncertainty associated with the count rate, ΔP the uncertainty in emission probability as listed in nuclear data tables, Δε the uncertainty in detection efficiency, and Δm the weighing uncertainty. The minimum detectable activity (MDA) for each radionuclide was calculated for the detector using Equation 3 (24, 25).


$$\begin{array}{l} MDA\;\left(Bq/kg\right)=\frac{\sigma \;\sqrt{B}}{\varepsilon .\;I_{\gamma }.t.m}\; \end{array}$$ (3)

where σ is the statistical coverage factor (1.645 at a 95% confidence level), B is the background count area at the relevant gamma energy, ε is the efficiency of the detector for that energy, I_γ_ represents the gamma emission probability, t is the counting duration in seconds, and m is the sample mass in kilograms.

To validate the measurement system's accuracy, the licensed soil reference material (IAEA-375) was analyzed. The recovery rates for the radionuclides examined in this study were found to be within 93–98%, confirming the reliability of the results.

### 2.3 Assessment of radiological hazards

The radium equivalent activity (Ra_eq_) is a widely applied index for assessing the radiological effects of stone samples containing different concentrations of natural radionuclides. This value provides a single index that accounts for the combined radioactivity of ^226^Ra, ^232^Th, and ^40^K, based on their relative contributions to gamma radiation exposure. The calculation assumes that 370 Bq/kg of ^226^Ra, 259 Bq/kg of ^232^Th, and 4.180 Bq/kg of ^40^K produce the same level of gamma radiation. Equation 4 was used in calculating the Ra_eq_ value (26).


$$\begin{array}{l} Ra_{\text{eq}}=A_{\text{Ra}}+1.43\times A_{\text{Th}}+0.077\times A_{\text{K}} \end{array}$$ (4)

where A_Ra_, A_Th_, and A_K_ represent the activity concentrations (in Bq/kg) of ^226^Ra, ^232^Th, and ^40^K, respectively. The internal hazard index (H_ex_), which represents external radiation exposure from ^226^Ra, ^232^Th, and ^40^K, can be calculated using Equation 5 in accordance with the model proposed by Krieger (26).


$$\begin{array}{l} H_{\text{in}}=A_{\text{Ra}}/185+A_{\text{Th}}/259+A_{\text{K}}/4,810 \end{array}$$ (5)

where the activity concentrations (Bq/kg) of ^226^Ra, ^232^Th, and ^40^K are denoted as A_Ra_, A_Th_, and A_K_, respectively. To ensure radiological safety, the value of internal hazard index (H_in_) must remain below unity to indicate the absence of significant risk to human health. The representative level index (Iγ), which serves to estimate gamma radiation risks from natural radionuclides, is obtained using Equation 6 (27).


$$\begin{array}{l} I_{\text{γ}}=A_{\text{Ra}}/150+A_{\text{Th}}/100+A_{\text{K}}/1,500 \end{array}$$ (6)

where A_Ra_, A_Th_, and A_K_ denote the activity concentrations (Bq/kg) of ^226^Ra, ^232^Th, and ^40^K, respectively. The European Commission (27) recommends reference limits of 300 Bq/kg, 200 Bq/kg, and 3,000 Bq/kg for these radionuclides, which correspond to an annual effective dose of 1 mSv (27). The representative level index (I_γ_) indicates how much external gamma radiation from building materials contributes to the yearly effective dose. For safety, the calculated I_γ_ value of a sample should not exceed 6; otherwise, the annual effective dose would surpass the 1 mSv/year limit established for the general population. The absorbed dose rate (D), expressed in nGy/h in air at a height of 1 m above ground level, was determined using Equation 7 and derived from the activity concentrations of ^226^Ra, ^232^Th, and ^40^K (12).


$$\begin{array}{l} D=0.462\times A_{\text{Ra}}+0.604\times A_{\text{Th}}+0.042\times A_{\text{K}} \end{array}$$ (7)

where A_Ra_, A_Th_, and A_K_ represent the activity concentrations (Bq/kg) of ^226^Ra, ^232^Th, and ^40^K, respectively. The annual effective dose (AEDE) was calculated using Equation 8 in order to evaluate the health implications of the absorbed dose rates.


$$\begin{array}{l} AEDE\left(mSv/y\right)=D\times DCF\times OF\times T \end{array}$$ (8)

where D denotes the absorbed dose rate in air (nGy/h), DCF is the dose conversion factor (0.7 Sv/Gy), OF represents the indoor occupancy factor (0.8), and T corresponds to the annual exposure time (8,760 h/y) (13). The annual gonadal dose equivalent (AGDE) is calculated using Equation 9 to evaluate the potential effects of ^226^Ra, ^232^Th, and ^40^K on important organs, including the gonads, bone marrow, and bone cells.


$$\begin{array}{l} AGDE\left(\mu Sv/y\right)=3.09\times A_{\text{Ra}}+4.18\times A_{\text{Th}}+0.314\times A_{\text{K}} \end{array}$$ (9)

where A_Ra_, A_Th_, and A_K_ represent the activity concentrations (Bq/kg) of ^226^Ra, ^232^Th, and ^40^K, respectively. The lifetime cancer risk associated with chronic exposure to gamma radiation from building materials is estimated using Equation 10.


$$\begin{array}{l} ELCR=AED\times DL\times RF \end{array}$$ (10)

where DL represents the duration of exposure (assumed to be 78.1 years), and RF is the risk factor for radiation-induced cancer, generally taken as 0.05 per Sv (28). This calculation provides an integrated measure of the potential additional cancer risk over an individual's lifetime. The world average value of LCR is 2.9 × 10^−3^ (29).

### 2.4 Statistical analyses

The statistical analysis and statistical graphics of the radioactivity results determined in the Bayburt Stone samples were performed using OriginPro (Version 9.0, OriginLab Corporation, Massachusetts, USA) and SPSS (Version 23.0, IBM corporation, New York, USA) software. The distributions of the ^226^Ra, ^232^Th, and ^40^K concentrations were described using some basic statistics. Whether the data conform to a normal distribution was analyzed using the Shapiro–Wilk normality test. Moreover, Pearson's correlation coefficient analysis was carried out to determine correlations between the radionuclides (30).

## 3 Results

Natural radioactivity concentrations of ^226^Ra, ^232^Th, and ^40^K were determined in 36 samples, comprising 30 collected from five mining sites and six obtained from the Bayburt stone factory in Bayburt, Türkiye. The results are presented in Table 1. Samples from the mining sites were coded as A1–A6, B1–B6, C1–C6, D1–D6, and E1–E6, while the end-user samples from the factory were designated as F1–F6.

**Table 1:** Radioactivity concentrations of the Bayburt stone samples.

Sample code	Collection point	Radioactivity concentration (Bq/kg)		
^226^Ra	^232^Th	^40^K
A1	ES	9.49 ± 1.07	2.57 ± 0.63	ND
A2	EUS	5.28 ± 0.81	2.03 ± 0.59	ND
A3	MS	6.82 ± 0.87	1.01 ± 0.33	16.96 ± 3.92
A4	MUS	2.21 ± 0.46	1.13 ± 0.31	13.14 ± 2.96
A5	LS	2.35 ± 0.58	1.87 ± 0.34	ND
A6	LUS	1.80 ± 0.45	1.23 ± 0.33	ND
B1	ES	2.06 ± 0.91	1.96 ± 0.42	10.31 ± 2.64
B2	EUS	2.56 ± 0.62	1.85 ± 0.36	ND
B3	MS	2.22 ± 0.68	2.00 ± 0.40	11.44 ± 2.45
B4	MUS	1.56 ± 0.55	2.16 ± 0.48	ND
B5	LS	2.44 ± 0.67	1.62 ± 0.41	19.86 ± 2.89
B6	LUS	1.88 ± 0.69	2.58 ± 0.60	15.21 ± 3.60
C1	ES	3.20 ± 0.91	1.57 ± 0.46	ND
C2	EUS	2.50 ± 0.80	1.54 ± 0.41	ND
C3	MS	2.66 ± 0.71	1.41 ± 0.42	ND
C4	MUS	1.93 ± 0.65	1.61 ± 0.38	ND
C5	LS	2.78 ± 0.71	1.54 ± 0.50	ND
C6	LUS	2.02 ± 0.68	1.83 ± 0.34	ND
D1	ES	2.51 ± 0.70	1.76 ± 0.62	17.03 ± 4.29
D2	EUS	3.98 ± 0.72	2.12 ± 0.34	ND
D3	MS	2.68 ± 0.72	1.11 ± 0.39	17.94 ± 2.37
D4	MUS	4.09 ± 0.70	1.05 ± 0.38	ND
D5	LS	2.43 ± 0.81	2.63 ± 0.61	19.81 ± 3.83
D6	LUS	2.42 ± 0.80	2.27 ± 0.39	15.67 ± 2.51
E1	ES	14.90 ± 1.66	15.87 ± 1.17	368.01 ± 17.96
E2	EUS	17.47 ± 1.47	18.84 ± 1.17	253.18 ± 18.68
E3	MS	13.56 ± 1.93	16.51 ± 1.79	483.22 ± 18.53
E4	MUS	14.76 ± 1.41	23.69 ± 1.14	368.66 ± 20.14
E5	LS	23.54 ± 1.98	19.73 ± 1.63	472.71 ± 24.31
E6	LUS	17.51 ± 1.48	11.44 ± 1.44	436.50 ± 20.07
F1	Factory	12.48 ± 1.77	10.58 ± 1.22	505.35 ± 16.77
F2	Factory	4.09 ± 0.85	1.54 ± 1.41	ND
F3	Factory	1.79 ± 0.43	1.67 ± 0.34	ND
F4	Factory	1.58 ± 0.49	0.53 ± 0.21	ND
F5	Factory	2.65 ± 0.97	1.24 ± 0.43	ND
F6	Factory	2.14 ± 0.71	2.18 ± 0.96	ND

A, Mining Site 1; B, Mining Site 2; C, Mining Site 3; D, Mining Site 4; E, Mining Site 5; F, Stone Factory; ES, Surface sampling at the entrance of the mine; EUS, Subsurface sampling at the entrance of the mine; MS, Surface sampling at the middle of the mine; MUS, Subsurface sampling at the middle of the mine; LS, Surface sampling at the end of the mine; LUS, Subsurface sampling at the end of the mine; ND, Not detected.

The minimum detectable activity (MDA) values for the high-purity germanium detector used in this study were determined to be 0.24 Bq/kg, 0.21 Bq/kg, and 2.49 Bq/kg for ^226^Ra, ^232^Th, and ^40^K, respectively. Although ^226^Ra and ^232^Th were detected in all Bayburt stone samples, ^40^K concentrations were not detected in 53% of the samples (19 samples).

The radioactivity concentrations in stone samples investigated in various countries for comparison with literature studies are given in Table 2.

**Table 2:** Comparison between radioactivity concentrations of building materials in this study and those of other countries.

Country	Sample type	Radioactivity concentration (Bq/kg)			References
		^226^Ra	^232^Th	^40^K	
Türkiye	Bayburt stone	5.56	4.62	179.12	This study
Türkiye	Marble	20.41	28.92	263.66	(17)
Türkiye	Ceramic	36.59	51.23	420.81	(33)
Egypt	Granit	31.2	57.8	1055.7	(34)
Cameroon	Brick	40	83	111	(35)
Iraq	Limestone	16.8	13.5	352.6	(36)
Slovakia	Building stone	8.82	25.05	364.16	(37)
Italy	Tuff	67	80	1,414	(38)
Italy	Lavic stone	438	93	2,163	(38)
Italy	Silt stone	17	16	209	(38)

The radiological risks associated with the natural radionuclides in the samples were assessed by calculating multiple radiological indices, including the radium equivalent activity (Ra_eq_), representative level index (I_γ_), internal hazard index (H_in_), absorbed dose rate (D), annual effective dose (AEDE), annual gonadal dose equivalent (AGDE), and lifetime cancer risk (LCR). Table 3 summarizes the calculated Ra_eq_, I_γ_, H_in_, D, AEDE, AGDE, and LCR values for the samples collected from both the mining sites and the stone factory.

**Table 3:** The calculated values of radiological hazard parameters for the Bayburt stone samples.

Sample code	Ra_eq_ (Bq/kg)	I_γ_	H_in_	D (nGy/h)	AEDE (μSv/y)	AGDE (μSv/y)	ELCR ( × 10^−5^)
A1	13.17	0.09	0.06	5.94	29.12	40.07	11.37
A2	8.18	0.06	0.04	3.67	17.98	24.80	7.02
A3	9.57	0.07	0.04	4.47	21.94	30.62	8.57
A4	4.84	0.03	0.02	2.26	11.06	15.68	4.32
A5	5.02	0.03	0.02	2.22	10.87	15.08	4.24
A6	3.56	0.02	0.01	1.57	7.72	10.70	3.02
B1	5.66	0.04	0.02	2.57	12.60	17.80	4.92
B2	5.21	0.04	0.02	2.30	11.28	15.64	4.41
B3	5.96	0.04	0.02	2.71	13.31	18.81	5.2
B4	4.65	0.03	0.02	2.03	9.94	13.85	3.88
B5	6.29	0.05	0.02	2.94	14.42	20.55	5.63
B6	6.74	0.05	0.02	3.07	15.04	21.37	5.87
C1	5.45	0.04	0.02	2.43	11.90	16.45	4.65
C2	4.70	0.03	0.02	2.09	10.23	14.16	3.99
C3	4.68	0.03	0.02	2.08	10.21	14.11	3.99
C4	4.23	0.03	0.02	1.86	9.14	12.69	3.57
C5	4.98	0.03	0.02	2.21	10.86	15.03	4.24
C6	4.64	0.03	0.02	2.04	10.00	13.89	3.91
D1	6.34	0.05	0.02	2.94	14.41	20.46	5.63
D2	7.01	0.05	0.03	3.12	15.30	21.16	5.98
D3	5.65	0.04	0.02	2.66	13.06	18.55	5.10
D4	5.59	0.04	0.03	2.52	12.38	17.03	4.83
D5	7.72	0.06	0.03	3.54	17.38	24.72	6.79
D6	6.87	0.05	0.03	3.15	15.44	21.89	6.03
E1	65.93	0.50	0.22	31.93	156.61	227.93	61.16
E2	63.91	0.47	0.22	30.08	147.58	212.23	57.63
E3	74.38	0.58	0.24	36.53	179.21	262.64	69.98
E4	77.02	0.58	0.25	36.61	179.6	260.39	70.13
E5	88.15	0.67	0.30	42.65	209.21	303.64	81.69
E6	67.48	0.52	0.23	33.33	163.52	238.99	63.85
F1	66.52	0.53	0.21	33.38	163.75	241.47	63.95
F2	6.29	0.04	0.03	2.82	13.83	19.08	5.40
F3	4.18	0.03	0.02	1.84	9.01	12.51	3.52
F4	2.33	0.02	0.01	1.05	5.13	7.07	2.00
F5	4.42	0.03	0.02	1.97	9.68	13.37	3.78
F6	5.26	0.04	0.02	2.31	11.31	15.73	4.42

## 4 Discussions

### 4.1 Radioactivity concentrations

The activity concentrations ranged from 1.56 ± 0.55 Bq/kg (sample B4, second mining site) to 23.54 ± 1.98 Bq/kg (sample E5, fifth mining site) for ^226^Ra; from 0.53 ± 0.21 Bq/kg (sample F4, factory) to 23.69 ± 1.14 Bq/kg (sample E4, fifth mining site) for ^232^Th; and from 10.31 ± 2.64 Bq/kg (sample B1, second mining site) to 505.35 ± 16.77 Bq/kg (sample F1, factory) for ^40^K. The average activity concentrations calculated across all 36 samples were 5.56 ± 0.90 Bq/kg for ^226^Ra, 4.62 ± 0.65 Bq/kg for ^232^Th, and 179.12 ± 9.88 Bq/kg for ^40^K, as summarized in Table 4, which presents the basic statistical parameters of the measured radioactivity concentrations.

**Table 4:** Some basic statistics of radioactivity concentrations in the Bayburt stone samples.

Code of the mining site	Number of sample	Average of radioactivity concentration (Bq/kg)		
		^226^Ra (min–max)	^232^Th (min–max)	^40^K (min–max)
A	6	4.66 ± 0.71 (1.80–9.49)	1.64 ± 0.42 (1.01–2.57)	15.05 ± 3.44 (13.14–16.96)
B	6	2.12 ± 0.69 (1.56–2.56)	2.03 ± 0.45 (1.62–2.58)	14.21 ± 2.90 (10.31–19.86)
C	6	2.52 ± 0.74 (1.93–3.20)	1.58 ± 0.42 (1.41–1.83)	ND
D	6	3.02 ± 0.74 (2.42–4.09)	1.82 ± 0.46 (1.05–2.63)	17.61 ± 3.25 (15.67–19.81)
E	6	16.96 ± 1.66 (13.56–23.54)	17.68 ± 1.39 (11.44–23.69)	397.05 ± 19.95 (253.18–483.22)
F^*^	6	4.12 ± 0.87 (1.58–12.48)	2.96 ± 0.76 (0.53–10.58)	505.35 ± 16.77 (ND−505.35)

^*^Stone factory (not a mining site); ND, Not detected.

For comparison, the global average activity concentrations in building materials are commonly reported as 50 Bq/kg for ^226^Ra, 50 Bq/kg for ^232^Th, and 500 Bq/kg for ^40^K (31). The activity concentrations determined in the Bayburt stone samples were generally lower than these reference values, except for the ^40^K concentration measured in sample F1. Based on activity concentration values alone, these findings indicate relatively low natural radioactivity levels in the studied material.

**Figure 7:**
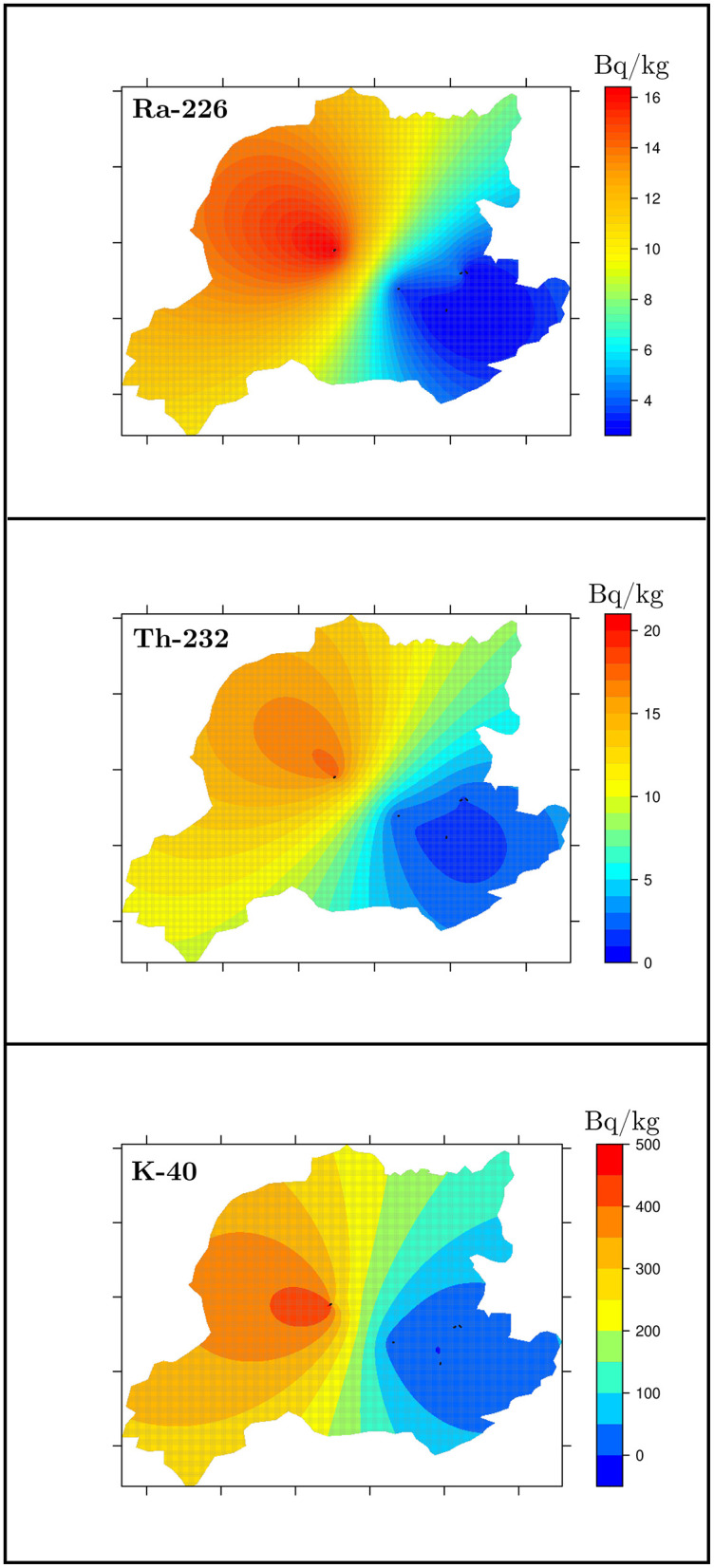
Distribution maps of the radioactivity concentrations in the Bayburt stone samples.

The spatial distribution of natural radioactivity levels in Bayburt stone samples collected from the five mining sites is illustrated in Figure 7. The distributions of ^226^Ra, ^232^Th, and ^40^K exhibit similar spatial patterns, with comparatively higher activity concentrations observed in the western and northwestern parts of the study area and lower values toward the eastern region. This variation likely reflects differences in geological formations, rock types, and mineral composition across the area (32).

To provide a national context, the measured activity concentrations were compared with similar studies conducted in Türkiye. Commercially sold marble samples reported mean activity concentrations of ^226^Ra = 20.41 Bq/kg, ^232^Th = 28.92 Bq/kg, and ^40^K = 263.66 Bq/kg (17), while ceramic samples from different areas exhibited mean values of ^226^Ra = 36.59 Bq/kg, ^232^Th = 51.23 Bq/kg, and ^40^K = 420.81 Bq/kg (33). In comparison, the Bayburt stone samples show lower mean concentrations of ^226^Ra, ^232^Th, and ^40^K. These differences may be attributed to variations in geological origin, mineralogical composition, heavy mineral content, and rock-forming processes specific to each region.

Following the national comparison, an international evaluation was performed. Table 2 presents a comparison of radioactivity concentrations in stone samples investigated in various countries. The data demonstrates that activity concentrations in building materials vary considerably across geographic regions, emphasizing the influence of geological and mineralogical factors on natural radionuclide content. As indicated in Table 4, the mean activity concentrations measured in Bayburt stone are generally lower than those reported in most international studies, apart from the ^40^K concentration determined in brick and siltstone samples from Cameroon and Italy, respectively. These comparisons further highlight the role of geographical origin in determining radionuclide concentrations in construction materials.

### 4.2 Assessment of the radiological hazard risks

The calculated Ra_eq_ values for the investigated Bayburt stone samples ranged from 2.33 Bq/kg to 88.15 Bq/kg, which are all below the recommended safety limit of 370 Bq/kg for building materials (12).

The minimum and maximum values of H_in_ as shown in Table 3 were found to be 0.01 and 0.30 respectively. This is an indication that the calculated H_in_ values for the Bayburt stone samples are less than one which implies that the stone are safe for used in construction.

The I_γ_ values ranged 0.02–0.67 with an average value of 0.14 as can be seen in Table 3. However, all computed values of I_γ_ are less than 6 which signify that the stones do not have the potentiality to pose consequential radiological danger to the community.

The assessed absorbed dose rate (D) values for the stone samples range from 1.05 to 42.65 nGy/h. The value of D in all the Bayburt stone samples is lower than the average global value of 84 nGy/h for indoor absorbed gamma rates (12).

The annual effective dose (AEDE) values generated as shown in Table 3, column 6 for the Bayburt stone samples vary from 5.13 to 209.21 μSv/y with an average value of 43.72 μSv/y. This average value is quietly less than the 1,000 μSv/y (1 mSv/y) recommended value as set by the European Commission (27). The estimated AEDE values indicate that the analyzed stone samples in this study could be used in constructions and streets without constituting a radiological hazard to the public.

The presented values in column 7 of Table 3 indicate the lifetime cancer risk (LCR) associated with the Bayburt stone samples. The calculated values range from 2.00 × 10^−5^ to 81.69 × 10^−5^ with an average of 17.07 × 10^−5^. The global average value of LCR is reported to be 2.90 × 10^−3^ (12). The calculated values of LCR for the Bayburt stone samples are quietly lower than the global average value. Therefore, it can be concluded that the evaluated samples would not present any significant radiological risk.

### 4.3 Evaluation of statistical analyses

**Figure 8:**
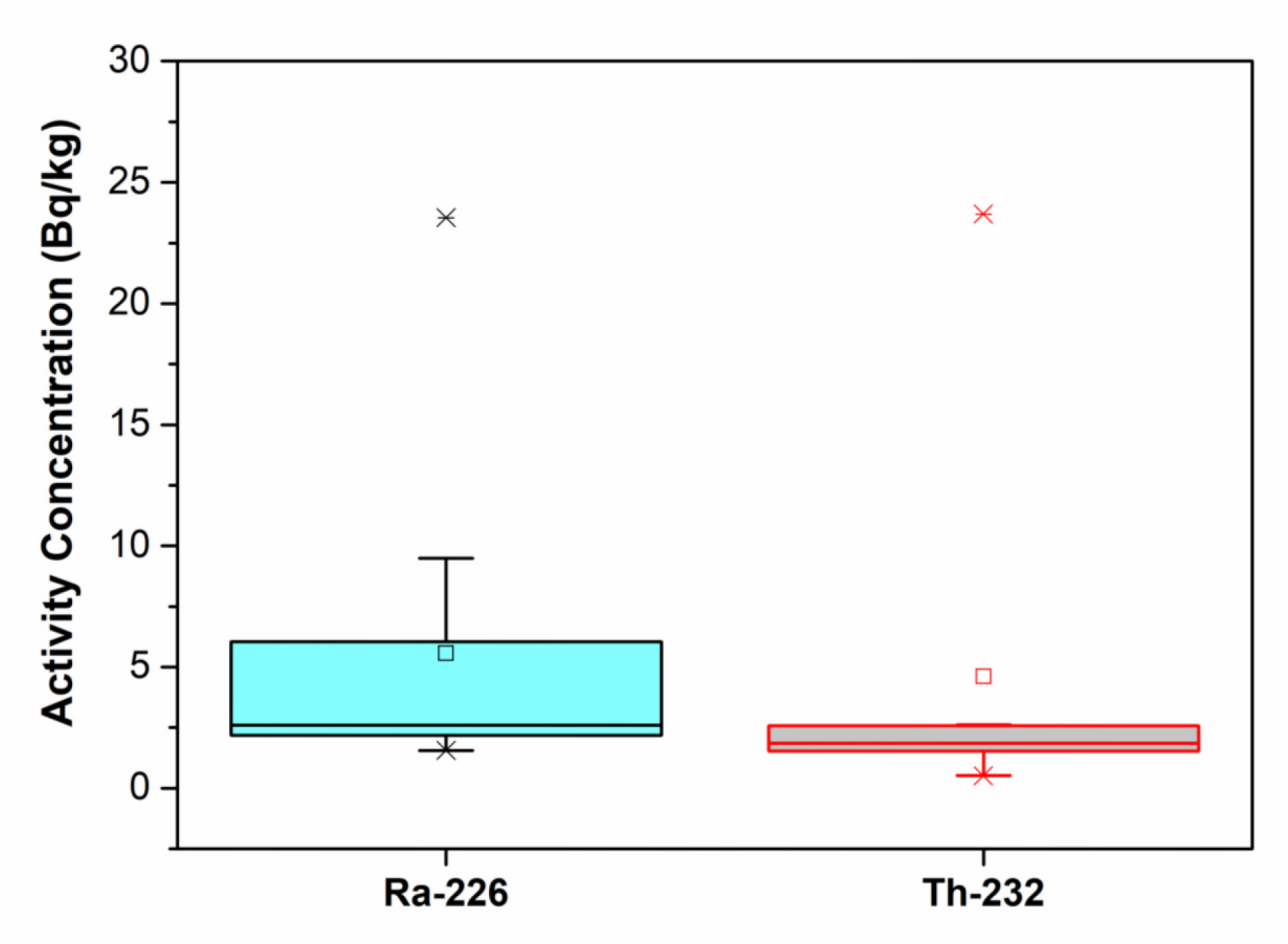
Statistical box chart of ^226^Ra and ^232^Th radioactivity concentrations in the Bayburt stone samples.

**Figure 9:**
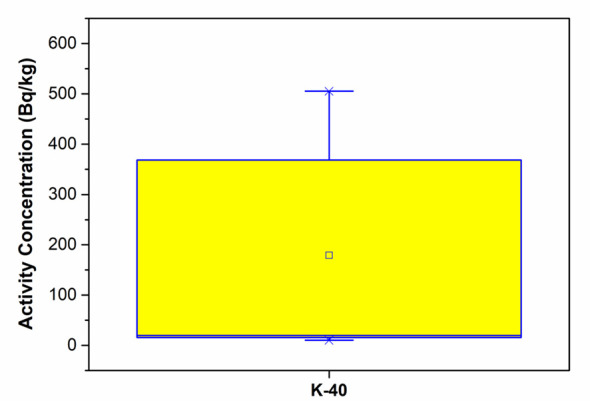
Statistical box chart of ^40^K radioactivity concentrations in the Bayburt stone samples.

Some basic statistics about radioactivity concentrations in Bayburt stone samples based on the mining sites were given in Table 4. An examination of Table 4 reveals that the highest activity averages for ^226^Ra and ^232^Th radioisotopes were found in samples belonging to the E-coded mining site. For the ^40^K radioisotope, the highest average activity was in samples from the stone factory. Statistical box charts of ^226^Ra, ^232^Th, and ^40^K radioactivity concentrations in the Bayburt stone samples were given in Figures 8, 9. As can be seen from the graphs, there are slight differences between the median and mean values. This change in ^226^Ra, ^232^Th, and ^40^K radioisotopes in the Bayburt stone samples may be due to processes such as secondary mineral formations (e.g., feldspar) or terrestrial contamination.

The Shapiro–Wilk normality test results showed that the ^226^Ra, ^232^Th, and ^40^K data were found not to follow a normal distribution. Differences between two groups were analyzed using the Mann–Whitney *U* test, and comparisons among three or more groups were performed using the Kruskal–Wallis *H* test. The identified groups were the mining sites, surface and subsurface sampling, and the entrance, middle, and exit points of the mining site. The results of the statistical tests indicated that radioactivity levels did not differ significantly between surface and subsurface samples (*p* > 0.05). Similarly, the radioactivity levels of samples from the entrance, middle, and exit points of the mines did not differ significantly (*p* > 0.05). For Radium-226, Thorium-232 and Potassium-40 radioisotopes, statistically essential differences (*p* < 0.05) were found between the radioactivity concentrations in the stone samples according to the mining sites (A, B, C, D, E). Furthermore, it is determined which mining sites had statistically significant differences, and consequently, no significant differences in radioactivity were found between mining sites A, B, C and D (*p* > 0.05), but a crucial difference in radioactivity existed between mining sites A, B, C, and D, and mining site E (*p* < 0.05).

**Figure 10:**
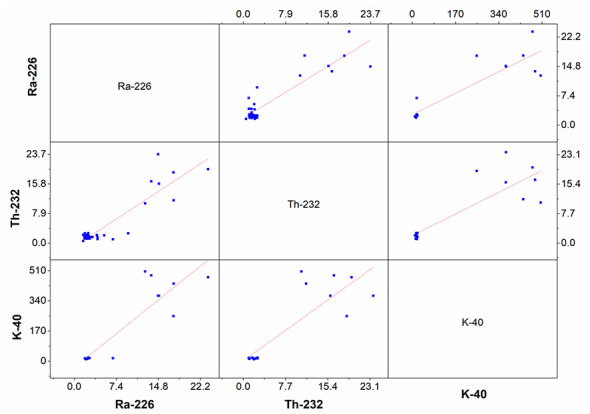
Statistical scatter matrix of the examined radioisotopes in the Bayburt stone samples.

Pearson correlation statistical analysis was performed in the form of a matrix to determine the relationship levels between ^226^Ra, ^232^Th, and ^40^K radioisotopes in Bayburt stone samples. The results of this statistical analysis for the radioactivity concentrations in the Bayburt stone samples are given graphically in Figure 10. It is clearly evident in Figure 10 that there is a positive and strong correlation between ^226^Ra, ^232^Th, and ^40^K radioisotopes. The correlation coefficients between ^226^Ra-^232^Th, ^226^Ra-^40^K, and ^232^Th-^40^K radioisotopes were found to be 0.82, 0.80, and 0.72, respectively. This strong relationship between the radioisotopes examined indicates that they are all of natural origin and have a relationship with each other underground.

## 5 Conclusions

The natural radionuclides concentrations of ^226^Ra, ^232^Th, and ^40^K in 36 Bayburt stone samples collected from five quarries and one natural stone factory in Bayburt Province, Türkiye, were analyzed using high-purity germanium (HPGe) spectrometry. Also, some radiological hazard parameters (Ra_eq_, H_in_, I_γ_, D, AEDE, AGDE, and ELCR) were calculated for each of the Bayburt stone samples and were compared with the values recommended by international organizations. The findings revealed that the mean radioactivity concentrations and radiological hazard parameters obtained in this study were below the corresponding global average values. The results for the analyzed Bayburt stone samples indicate significant variations in natural radionuclide concentrations among stone products sourced from different mines. The results suggest that Bayburt stone samples can be safely utilized in construction without posing notable radiological dangers to human well-being in any environment. The results of the current study have the potential to guide future studies.

## Data Availability

The raw data supporting the conclusions of this article will be made available by the authors, without undue reservation.
